# Is there a critical LH level for hCG trigger after the detection of LH surge in modified natural frozen-thawed single blastocyst transfer cycles?

**DOI:** 10.1007/s10815-020-01974-5

**Published:** 2020-10-14

**Authors:** Semra Kahraman, Yucel Sahin

**Affiliations:** Assisted Reproductive Technologies and Reproductive Genetics Center, Istanbul Memorial Hospital, Piyalepasa Bulvari, Sisli, 34384 Istanbul, Turkey

**Keywords:** mNC-FET, LH value, hCG day

## Abstract

**Purpose:**

There is no consensus yet in the literature on an optimal luteinizing hormone (LH) level for human chorionic gonadotrophin (hCG) trigger timing in patients undergoing frozen-thawed embryo transfer (FET) with modified natural cycles (mNC). The objective of our study was to compare the clinical results of hCG trigger at different LH levels in mNC-FET cases.

**Methods:**

This retrospective study was conducted in Istanbul Memorial Hospital ART and Genetics Center. A total of 1076 cases with 1163 mNC-FET cycles were evaluated. LH levels between the start of LH rise (15 IU/L) and LH peak level (> 40 IU/L) were evaluated. Cycles were analyzed in four groups: group A (*n* = 287) LH level on the day prior to the day of hCG; groups B, C and D, LH levels on the day of hCG: group B (*n* = 245) LH 15–24.9; group C (*n* = 253), LH 25–39.9; group D (*n* = 383) LH ≥ 40. Cycle outcomes in the four groups were compared.

**Results:**

Subgroup analyses of mNC-FET groups showed that implantation, clinical and ongoing pregnancy rates, and pregnancy losses were not significantly different in patients with different LH levels on the day of hCG trigger.

**Conclusion:**

Our study suggests that hCG can be administered at any time between the start of LH rise (≥ 15 IU/L) and LH peak level (≥ 40 IU/L) without a detrimental effect on clinical outcome.

## Introduction

Advances in cryopreservation techniques have resulted in a dramatic increase in freeze-all cycles. Reasons for this preference include the higher rate of viable embryos available after thawing and the near elimination of the risk of ovarian hyperstimulation syndrome (OHSS). Furthermore, many studies have shown increased implantation rates when transfer takes place in a more natural uterine environment without elevated levels of hormones [1–4].

Appropriate endometrial preparation therefore plays an essential role in successful implantation in FET cycles. The two main alternative approaches for endometrial preparation are estrogen and progesterone replacement treatment (artificial cycle) or the use of natural cycle, depending on the regularity or pattern of the menstrual cycle. Because of possible adverse hormonal effects, including a higher risk of thrombo-embolic events, and because of the inconvenience to patients, in our center, the artificial cycle is used only for cases with anovulatory cycles and/or endometrial thinning.

In natural cycle, the alternatives are true and modified natural cycles. True natural cycle (tNC)-FET requires strict hormonal and follicular development monitoring. On the other hand, the mNC-FET has a number of advantages. Firstly, continual hormonal monitoring is not required. Secondly, once the hCG trigger is performed, the timing of embryo transfer can be determined. Thirdly and importantly, hCG injection not only triggers ovulation but also supports the luteal phase. Because of these advantages, mNC-FET is the preferred technique in our center. However, the results of studies focusing on LH level at the time of hCG in mNC-FET cycle have been variable and there is no consensus yet on the optimal LH level for hCG trigger timing.

There are several papers examining the optimal identification of LH surge and peak and the determination of embryo transfer day. Irani et al. [5] showed that in true natural cycle, defining the surge as occurring on the day of an LH of ≥ 17 IU/L with a drop of ≥ 30% in estradiol (E2) levels, the following day was associated with better NC-FET outcomes than defining the surge as the day representing the highest serum LH level with a ≥ 30% drop in E2 levels on the same day [5]. A further related study by Reichman et al. [6], in which the LH surge was defined by an LH level > 17 IU/L during the follicular phase with a subsequent dropping E2 level thereafter, also reported improved cycle outcomes with hCG booster given to patients within 1 day post-LH surge. Bartels et al. [7] reported that when LH reached ≥ 20 IU/L, there was no difference in outcomes regardless of whether transfer was performed on day 6 or day 7 after the initial surge. On the other hand, Litwicka et al. [8] suggested that LH elevation ≥ 13 before hCG administration may negatively affect the clinical results in mNC and advised routine performance of LH determination during the regular ultrasonographic evaluation of dominant follicle growth and avoidance of hCG in cases of elevated LH, when embryo transfer should be performed after spontaneous follicular rupture. Kyrou et al. [9] reported higher ongoing pregnancy rates in patients with spontaneous LH surge compared with hCG-administered patients undergoing natural cycle intrauterine insemination. They suggested that ovulation and implantation are driven primarily by the LH surge itself and only to a lesser extent by exogenous hCG [9]. Another study by Fatemi et al. [10] concluded that there is emerging evidence that the administration of hCG in the late follicular phase induces a cascade of events in the endometrium, which would have started several days later in the presence of endogenous LH rise and that these events appear to have a negative impact on ongoing pregnancy rate.

Our retrospective study, based on 1168 mNC-FET cycles in good prognosis patients only, investigates whether there is a critical LH level for hCG trigger after the detection of LH surge in modified natural frozen-thawed single blastocyst transfer cycles.

## Material and methods

### Patient population

This retrospective study, conducted between January 2015 and September 2019 in Istanbul Memorial Hospital ART and Genetics Center was based on 1168 mNC-FET cycles in 1076 cases with regular menses and evidence of ovulation. Patients with regular menses (24–35 days) for at least the last 6 months and with ovulatory cycles proven with ultrasonography or with hormonal evaluation and who were undergoing frozen-thawed single blastocyst transfer cycles were evaluated. Inclusion criteria were: ≤ 42 years old, body mass index (BMI) < 30 kg/m^2^, blastocyst-stage frozen-thawed single embryo transfer on day 5, or only rarely on day 6 (*n* = 12), endometrial thickness of ≥ 8 mm. Cases with recurrent pregnancy losses (≥ 3) and repeated implantation failure (> 3 cycles) were excluded. Patients undergoing PGT-A were not included in this study. This study was approved by Istanbul Memorial Hospital institutional review board (Number: 2020/004).

Our aim was to compare single blastocyst transfer outcomes, therefore a good prognosis group was chosen for the study, because elective blastocyst-stage single embryo transfer is more likely to be possible. Furthermore, including only good prognosis patients excluded any possible bias which could have resulted from compromised clinical characteristics of patients. In cases with elevated serum progesterone (≥ 3 ng/ml) and/or the formation of corpus luteum, the cycle was continued as a true natural cycle and therefore not included in our study.

The following data were recorded for each patient: the causes of infertility (male factor, unexplained infertility, tubal factor, endometriosis, diminished ovarian reserve (DOR) and combined factors), maternal age, BMI, and anti-Mullerian hormone (AMH) level. Fresh cycle characteristics were evaluated, including mean number of cumulus oocyte complex (COC), metaphase II (MII) oocytes and fertilized oocytes, and blastocyst morphology (the percentage of excellent, good, moderate, poor).

### Follicular monitoring and endometrial preparation

Ultrasonographic evaluation was carried out on the second day of menstruation to rule out the presence of ovarian cysts or other pelvic pathologies. It was repeated on day 9 or 10, depending on the cycle length, to observe whether there was a spontaneously growing dominant follicle. After day 9 or 10, it was repeated every 2 or 3 days to monitor endometrial thickness and follicular size. When the follicle reached 15 mm, serum LH and estrogen were evaluated and levels were measured every 24 h until detecting the LH surge.

The duration from the first day of menstrual cycle to the hCG trigger day and then to the embryo transfer day**,** follicular diameter on hCG day, endometrial thickness on hCG day, E2 level on hCG day were recorded. Definitions of the beginning of LH surge vary between ≥ 10 IU/L and ≥ 20 IU/L in different studies [6, 7, 11–13]. In our study LH surge was defined as the LH level reaching ≥ 15 IU/L and LH peak as the LH level reaching ≥ 40 IU/L. This was determined by evaluating data based on the strict monitoring of estrogen, LH, and progesterone levels of true natural cycle cases in our clinic over a period of 8 years.

mNC-FET cycles were evaluated retrospectively and grouped according to LH levels. LH levels on the day of hCG were the main focus of the study. In groups B, C and D, LH levels on the day of hCG were: group B (*n* = 245) LH 15–24.9 IU/L; group C (*n* = 253), LH 25–39.9 IU/L; group D (*n* = 383) LH ≥ 40 IU/L. In these groups, recombinant hCG (rhCG) (Ovitrelle®, Merck Serono, Switzerland) was administered at any time between the start of LH rise (15 IU/L) and LH peak level (≥ 40 IU/L). rhCG was given in the evening in all groups and frozen embryo transfer was scheduled for 6 days after rhCG administration.

When our data was evaluated retrospectively, we identified a fourth group (group A, *n* = 287) who lived at significant distances from the hospital and for whom next day clinic attendance was not practical. Ultrasonographic evaluations had been continued until their final appointment at our clinic, when their LH levels (mean: 14.8 IU/L) indicated that LH rise had already started or would start the next day. Therefore, one more day was allowed either for the follicle to increase in size or for the LH level to rise. They were advised to administer the hCG trigger in the evening of the next day instead of returning to the hospital. Therefore, in this group, the LH level on the day prior to the day of hCG was recorded. The findings regarding this group are included in order to illustrate practical approaches for the clinician.

Frozen embryo transfer was scheduled for 5 or rarely 6 days after rhCG administration. Vaginal progesterone gel with 90 mg (8%) (Crinone® Merck Serono, Switzerland) administered once a day was started two days after rhCG administration to reduce the risk of luteal phase deficiency and poor endogenous luteal phase. When pregnancy occurred, the same daily doses of progesterone vaginal gel were continued until the 10th week of gestation.

### Embryo scoring, vitrification, and thawing

Blastocysts were scored before vitrification according to Gardner’s classification [14]. Good or top-quality blastocysts (at least 3BB) were vitrified on day 5 and day 6 morning with Kitazato vitrification media, using Cryotops® as carriers. Blastocysts were thawed with Kitazato warming media according to manufacturer’s instructions. Embryos were first checked for survival 30 min after thawing. A second check occurred 2 h after warming for re-expansion, hatching, extensive cytoplasmic granulation, and the presence of necrotic foci, which are predictors of the rates of implantation, pregnancy, and live birth [7]. Eligible blastocysts with at least 80% re-expansion and vitality were transferred in the afternoon of the same day.

### Evaluation of clinical outcomes

Nine days after blastocyst transfer, serum β-hCG was measured. Clinical pregnancy was diagnosed if gestational sac was observed, viable pregnancy was defined as the presence of fetal heart beat at week 7 and ongoing pregnancy was defined as a 12-week viable pregnancy.

All pregnancy rates including biochemical pregnancy rate (BPR), clinical pregnancy rate (CPR), and ongoing pregnancy rate (OPR) were calculated according to per embryo transfer cycle. BPR was defined as the number of biochemical pregnancies/number of ET cycles, CPR as the number of clinical pregnancies/number of ET cycles and OPR as the number of ongoing pregnancies/number of ET cycles.

Total pregnancy loses (TPL) was defined as pregnancies resulting in first trimester losses. Biochemical pregnancy loss (BPL) was defined as pregnancy resulting in loss before sonographically visible gestational sac formation and clinical pregnancy loss (CPL) was defined as pregnancy loss after gestational sac formation and before 12-week pregnancy.

The rate of biochemical pregnancy losses (BPLR) was defined as the number of BPL/number of biochemical pregnancies. The rate of clinical pregnancy losses (CPLR) was defined as the number of CPL/number of clinical pregnancies. The rate of total pregnancy losses (TPLR) was defined as the number of TPL/number of biochemical pregnancies.

### Statistics

In our study, the total number of cases needed to obtain 80% power at α = 0.03 level was found to be *n* = 787 cycles. However, to take account of possible losses, it was considered that a cycle number of approximately 1000 would be more reliable.

For statistical analyses NCSS (Number Cruncher Statistical System) 2007 Statistical Software (Utah, USA) program was used. Demographics of patients were reported as minimum, maximum, mean ± SD and 95% confidence interval of the mean. In addition, Kolmogorov Smirnov test and box plot charts were used to compare the data to normal distribution. As a result of these tests, it was determined that data showed a normal distribution for all variables. One-way ANOVA test with Bonferroni correction for multiple comparisons was applied for continuous variables and Pearson chi-square test for categorical group comparisons. A *p* value of *p* < 0.05 was accepted as statistically significant.

## Results

A total of 1076 cases with 1168 mNC-FET cycles were retrospectively evaluated.

Table 1 shows patient characteristics according to LH levels. There was no statistically significant difference between the LH groups in terms of demographic characteristics or infertility diagnosis. Bonferroni correction showed that the only significant differences occurred between group D and group B in terms of cycle lengths to hCG day (*p* = 0.027) and to embryo transfer day (*p* = 0.033). Cycle length from the first day of menstruation to hCG day was significantly longer in group D than in group B (*p* = 0.031) and also cycle length from the first day of menstruation to ET day was significantly longer in group D than in group B (*p* = 0.048).

**Table 1:** Patient and FET cycle characteristics according to LH levels

Characteristics	Group A (*n* = 287)	Group B (*n* = 245)	Group C (*n* = 253)	Group D (*n* = 383)	*p* value
Age (years) (mean ± SD)	32.87 ± 4.96	32.43 ± 4.82	32.4 ± 4.71	32.18 ± 4.81	^a^0.452
BMI (kg/m^2^) (mean ± SD)	23.99 ± 3.98	23.92 ± 4.02	24.42 ± 4.28	24.13 ± 4.01	^a^0.639
AMH (ng/ml) (mean ± SD)	3.11 ± 2.80	3.19 ± 2.55	3.16 ± 2.33	3.01 ± 2.36	^a^0.896
Cycle length up to hCG day (d) (mean ± SD)	13.05 ± 2.80	11.69 ± 2.17	13.16 ± 2.66	13.84 ± 2.95	^a^0.027*
Cycle length up to ET day (d) (mean ± SD)	19.08 ± 3.39	18.07 ± 2.95	19.11 ± 3.05	19.74 ± 3.44	^a^0.033*
Peak endometrial thickness (mm) (mean ± SD)	10.59 ± 1.70	10.73 ± 1.79	10.65 ± 1.84	10.89 ± 1.71	^a^0.055
E2 Level on hCG day (pg/ml) (mean ± SD)	238.28 ± 105	295.1 ± 100.5	287.3 ± 89.6	255 ± 84.8	^a^0.055
LH level on the day before hCG (group A) and on the day of hCG (groups B, C, and D) (IU/L) (mean ± SD)	14.8 ± 15.4	20.2 ± 12.8	32.1 ± 14.2	61.7 ± 18.9	^a^0.01*
Follicle size (mm) (mean ± SD)	18.3 ± 1.5	18.1 ± 1.4	18.4 ± 1.6	18.3 ± 1.5	^a^0.113
Infertility factor					^b^0.465
Male factor	72 (25.1)	64 (26.1)	69 (27.3)	99 (25.8)	
Unexplained	61 (21.2)	49 (20.1)	47 (18.6)	82 (21.4)	
Tubal factor	52 (18.2)	40 (16.3)	40 (15.7)	63 (16.4)	
Endometriosis	6 (2.1)	4 (1.6)	7 (2.8)	6 (1.6)	
DOR	44 (15.3)	45 (18.4)	43 (17)	59 (15.4)	
Combined factor	52 (18.1)	43 (17.5)	47 (18.6)	74 (19.4)	

^a^One-way ANOVA

^b^Pearson chi-square test

*p < 0.05

The differences between the groups were compared using the Bonferroni correction. Cycle length from the first day of menstruation to hCG day is significantly longer in group D than in group B (*p* = 0.031) and also cycle length from the first day of menstruation to ET day is significantly longer in group D than in group B (*p* = 0.048)

Table 2 summarizes the fresh cycle characteristics. No significant difference was observed between the groups.

**Table 2:** Fresh cycle characteristics of patients

Characteristics	Group A (*n* = 287)	Group B (*n* = 245)	Group C (*n* = 253)	Group D (*n* = 383)	*p* value
COC (mean ± SD)	13.07 ± 8.21	14.27 ± 9.36	13.65 ± 7.52	14.51 ± 8.16	^a^0.140
MII (mean ± SD)	11.14 ± 6.59	12.26 ± 8.05	11.75 ± 6.28	12.46 ± 6.96	^a^0.123
PN2 (mean ± SD)	9.40 ± 5.65	10.19 ± 6.83	9.7 ± 5.37	10.45 ± 5.93	^a^0.145
Blastocyst ET morphology					^b^0.893
Excellent (*n*, %)	176 (61.3)	147 (60)	150 (59.3)	230 (60.1)	
Good (*n*, %)	90 (31.4)	79 (32.2)	91 (36)	117 (30.5)	
Moderate (*n*, %)	16 (5.6)	15 (6.1)	9 (3.6)	27 (7)	
Poor (*n*, %)	5 (1.7)	4 (1.6)	3 (1.2)	9 (2.3)	

^a^One-way ANOVA

^b^Pearson chi-square test

*p* < 0.05

Table 3 shows pregnancy outcomes. No significant difference was observed between different LH groups in BPR, CPR, and OPRs. In addition, no significant difference was observed in BPLR, CPLR and TPLR between different LH groups.

**Table 3:** Pregnancy outcomes of patients

	Group A (*n* = 287)	Group B (*n* = 245)	Group C (*n* = 253)	Group D (*n* = 383)	*p* value
Biochemical pregnancies (*n*, %)	214 (74.6)	177 (72.2)	193 (76.3)	289 (75.5)	^b^0.867
Clinical pregnancies (*n*, %)	201 (70.0)	167 (68.2)	180 (71.1)	268 (70)	^b^0.908
Ongoing pregnancies (*n*, %)	183 (63.8)	155 (63.3)	165 (65.2)	248 (64.8)	^b^0.876
Biochemical pregnancy losses (*n*, %)	13 (6.1)	10 (5.6)	13 (6.7)	21 (7.3)	^b^0.445
Clinical pregnancy losses (*n*, %)	18 (8.9)	12 (7.2)	15 (8.3)	20 (7.5)	^b^0.907
Total pregnancy losses (*n*, %)	31 (14.5)	22 (12.4)	28 (14.5)	41 (14.2)	^b^0.886

^b^Pearson chi-square test

*p* < 0.05

## Discussion

Artificial endometrial preparation with hormones and natural cycle are the most commonly used endometrial preparation techniques [15]. The most effective, practical, convenient and safe endometrial preparation protocol is still under debate. The natural cycle may be preferable for women with regular menstrual cycles as, being more physiological, it requires less medication [16]. tNC-FET requires strict hormonal and follicular development monitoring. mNC-FET, which requires administration of exogenous hCG, has the advantage of supporting the luteal phase.

Transfer of the thawed embryo should be conducted at the time of optimal endometrial receptivity, which is determined by the time of LH surge during tNC-FET. In the mNC, in order to determine the timing of the hCG administration some studies report using follicular size only whereas others use both follicular size and the detection of LH surge [17–19]. Currently however, there is no consensus on the exact parameters that define the LH surge.

Groenewoud et al. [17] found no optimal cut-off LH concentration and argued that regular ultrasound evaluation of the dominant follicle in combination with hCG triggering of ovulation was an effective way of FET planning. They concluded that single LH determination prior to ovulation induction in unstimulated cycle FET does not seem to have added clinical value [17]. However, Saupstad et al. [20] reported that in a natural cycle the follicle size corresponding to the start of LH rise varies between different patients. Therefore, relying on follicular size only may result in the administration of hCG either too early or too late, consequently adversely affecting endometrial receptivity, thereby resulting in asynchronicity between the endometrium and the transferred blastocyst. In our study, the timing of hCG was determined not only by follicular size but after the confirmation of an LH rise. In cases where the LH rise occurred when the follicular diameter was only 14–15 mm, LH surge was accepted as the main criterion for hCG administration in groups B, C and D. Furthermore, E2 level was simultaneously monitored with LH, but only until hCG day, and E2 drop was not used as a criterion for hCG timing. This was in order to reduce the number of blood samples required from patients.

In Reichmann’s study, the LH surge was defined as an LH level > 17 IU/L during the follicular phase with a subsequent dropping E2 level thereafter. They hypothesized that hCG supplementation after the LH surge would support endogenous steroidogenesis leading to improved pregnancy outcomes. They concluded that natural cycle FET in which the luteal phase is buttressed with both a single hCG injection after the endogenous LH surge, as well as vaginal progesterone after transfer, are associated with higher clinical success rates with minimal negative impact on the patient experience [6]. Our strategy also resulted in high ongoing pregnancy rates (Table 3). According to our results, provided the follicular diameter and e2 levels are appropriate, hCG can be administered at any time between the start of LH rise (15 IU/L) and LH peak level (> 40 IU/L), without having a detrimental effect on ongoing pregnancy. Furthermore, there was a lower rate of clinical pregnancy losses (6.1%, 5.6%, 6.7%, 7.3%) when compared to our pregnancy losses rate in artificial cycle (17.2%) [21].

There have been several studies questioning the use of hCG trigger. Mansour et al. [22] reported that hCG increases endometrial progesterone receptors and its level is positively correlated with the level of trophoblastic tolerance. Weisman et al. [23] reported no differences in terms of FET outcome between spontaneous ovulatory cycle patients and hCG-administered patients in FET.

Three studies report a deleterious effect of hCG triggering on implantation and pregnancy rates in spontaneous LH rise patients [8–10]. Fatemi et al. [10] reported that in cryopreserved-thawed human embryo transfer, true natural cycle is superior to human chorionic gonadotropin-induced natural cycle. This study was terminated early, when a pre-specified interim analysis found a significantly higher ongoing pregnancy rate in the spontaneous LH group as compared with the hCG group (31.1% vs. 14.3%; difference 16.9%, 95% confidence interval 4.4–28.8%). However, Montagut et al. [24] criticized this study for a number of reasons. Firstly, some women in the mNC-FET group also had an LH rise on the day of hCG administration. Secondly, day 3 embryos were transferred. Furthermore, no luteal phase support was given in the mNC-FET group, which could have had a negative impact on clinical outcome [25, 26]. Finally, FET was scheduled after an identical time interval, irrespective of tNC or mNC-FET, even though ovulation may occur at a later stage after hCG administration compared to ovulation after spontaneous LH rise [25]. Fatemi et al. [10] concluded that there is emerging evidence that the administration of hCG in the late follicular phase induces a cascade of events in the endometrium, which would have started several days later in the presence of endogenous LH rise and that these events appear to have a negative impact on ongoing pregnancy rate. Using our strategy, the ongoing pregnancy rates were (63.8%, 63.3%, 65.2%, 64.8%), which are significantly higher than the OPR of 14.3% in their studies. Furthermore, Reichman et al. [6] reported an OPR of 70% in a retrospective cohort study of women who underwent natural cycle FET with transfer of a single euploid blastocyst and who received hCG 1 day post-LH surge. Thus, Fatemi et al.’s cascade hypothesis of events in the endometrium does not explain the low ongoing pregnancy rates that they reported.

Besides the main advantage of supporting the luteal phase in natural cycle via its effect on the corpus luteum, hCG also triggers ovulation and facilitates the programming of embryo transfer day. It is possible that hCG provides a signal to the endometrium regarding future blastocyst implantation, thus fostering the growth and differentiation of trophoblast cells, and the establishment of placental villous structures [27]. It is known that hCG biosynthesis begins early in embryo development, and higher concentrations are found in the uterine cavity prior to implantation. The exogenous administration of hCG during in vitro fertilization may mimic the local effects of hCG when fertilization occurs naturally [28]. Therefore, it is possible that, rather than being detrimental, as has been reported in some studies, the administration of hCG in mNC-FET may have a similar beneficial effect to endogenous hCG.

The protocol at our clinic includes the administration of vaginal progesterone 2 days after the administration of hCG. The question of whether the administration of progesterone to support the luteal phase is necessary for all patients undergoing mNC-FET is still under debate. However, the majority of studies suggest that administration of vaginal progesterone in the luteal phase appears to increase significantly the clinical pregnancy rate [29–31].

One limitation of the study is that it was a retrospective study of good prognosis patients only*.* It also included a group for whom our usual protocol regarding the timing of hCG was adjusted for the practical reasons described previously and whose LH levels were measured the day before hCG rather than on the day of hCG.

One of the strengths of our study is that it is a single center study, which decreases differences in practice. Furthermore, it is based on the high number of 1168 mNC-FET cycles. The administration of hCG at any time between the start of LH rise (15 IU/L) and peak level (> 40 IU/L) has a significant practical advantage in clinical practice. In the near future, it is likely that modified natural cycle will be used more widely in FET cycles; thus, prospective studies of patients undergoing FET cycles other than those who have good prognosis are needed to guide best practice.
